# Cattle gastrointestinal nematode egg-spiked faecal samples: high recovery rates using the Mini-FLOTAC technique

**DOI:** 10.1186/s13071-020-04107-0

**Published:** 2020-05-06

**Authors:** Alessandra Amadesi, Antonio Bosco, Laura Rinaldi, Giuseppe Cringoli, Edwin Claerebout, Maria Paola Maurelli

**Affiliations:** 1 Department of Veterinary Medicine and Animal Production, University of Naples Federico II, CREMOPAR, Naples, Italy; 2 Department of Virology, Parasitology and Immunology, Faculty of Veterinary Medicine, Ghent University, Ghent, Belgium

**Keywords:** Gastrointestinal nematodes, Cattle, Faecal Egg Count, Mini-FLOTAC, McMaster, egg-spiking, Belgium, Italy

## Abstract

**Background:**

Faecal egg count (FEC) techniques are commonly used to detect gastrointestinal nematodes (GINs) in cattle and to determine anthelmintic efficacy/resistance through the faecal egg count reduction test (FECRT). Mini-FLOTAC is one of the techniques recommended for a standardised FEC/FECRT of helminth eggs in cattle. However, only one paper evaluated the recovery rate of GIN eggs by Mini-FLOTAC (compared to McMaster and modified-Wisconsin method) in cattle, using only a level of contamination of 200 eggs per gram (EPG) of faeces and using GIN eggs collected from goat faeces to spike faecal samples from cattle. To further study the recovery rate of GIN eggs from cattle faeces, this study was conducted in two laboratories, one in Belgium and one in Italy to evaluate the sensitivity, accuracy, precision and reproducibility of the Mini-FLOTAC and McMaster techniques (at two reading levels: grids and chambers) for the detection of GIN eggs in spiked bovine faecal samples.

**Methods:**

In both countries, spiked cattle faecal samples with five different levels of egg contamination (10, 50, 100, 200 and 500 EPG) of GINs were used. The study was performed in both laboratories by the same expert operator and using the same standard operating procedures (SOPs) for the Mini-FLOTAC and McMaster techniques. Sensitivity, accuracy and precision were calculated for each technique and for each level of contamination. Statistical analyses were performed to evaluate differences in performance between the two techniques.

**Results:**

Mini-FLOTAC had a higher sensitivity (100% at all EPG levels for Mini-FLOTAC *vs* 0–66.6% for McMaster chambers and grids at levels< 100 EPG), a higher accuracy (98.1% mean value for Mini-FLOTAC *vs* 83.2% for McMaster grids and 63.8% for McMaster chambers) and a lower coefficient of variation (10.0% for Mini-FLOTAC *vs* 47.5% for McMaster grids and 69.4% for McMaster chambers) than McMaster. There was no significant difference in the recovery of GIN eggs between the two studies performed in Belgium and in Italy.

**Conclusions:**

The high GIN egg recovery rate detected by Mini-FLOTAC and the similar results obtained in Belgium and in Italy indicated that the diagnostic performance of a FEC technique was not dependent on the laboratory environment.
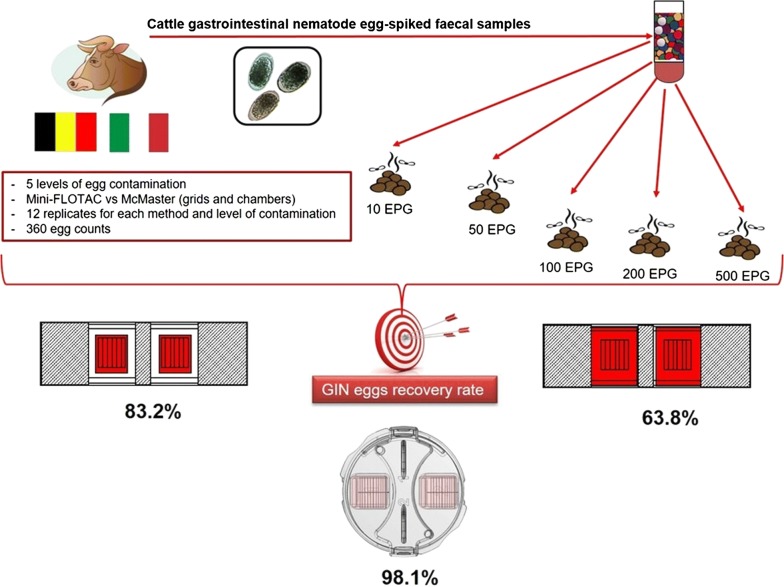

## Background

Gastrointestinal nematode (GIN) infections may negatively influence animal health, welfare and productivity in grazing cattle worldwide [1]. The negative impact of GIN on livestock farming is further exacerbated by the emergence of anthelmintic resistance (AR) in cattle nematodes [2–4].

In order to limit AR and the misuse/abuse of anthelmintics in cattle, the use of regular diagnostic testing is suggested as one of the options for a sustainable control strategy [5]. Diagnostic methods for GIN include faecal egg count (FEC) techniques that are commonly used in parasitological research and veterinary practice to indirectly assess GIN burdens and determine anthelmintic efficacy/resistance through the faecal egg count reduction test (FECRT) [6]. FEC techniques based on easy-to-use devices with high diagnostic performance in terms of sensitivity, accuracy, precision and reproducibility are suggested to perform reliable and exploitable FEC/FECRT in cattle [5, 7].

Mini-FLOTAC is considered a good candidate for a standardised FEC/FECRT of helminth eggs in livestock [8]. This method, in fact, has been compared with different diagnostic techniques, i.e. Cornell-Wisconsin, McMaster and FECPAK, and was shown to be more sensitive, accurate and precise for FEC and FECRT of GINs in sheep [7, 9–12]. Mini-FLOTAC has been also successfully used to perform FEC and FECRT (in the laboratory and on-farm) in cattle [5, 7, 13, 14]. However, only a single study by Paras et al. [7] evaluated the recovery rate of GIN eggs by Mini-FLOTAC (compared to McMaster and modified-Wisconsin) in cattle. The authors found an accuracy of 70.9%, but eggs used to spike samples were collected from goats and only one level of contamination (i.e. 200 eggs per gram of faeces, EPG) was used.

To further investigate the recovery rate of added GIN eggs from cattle, the present paper reports the findings of a study conducted in two laboratories, one in Belgium and one in Italy, to compare Mini-FLOTAC and McMaster (at two reading levels, i.e. grids and chambers) methods, in terms of sensitivity, accuracy, precision and reproducibility, using GIN egg-spiked faecal samples at five different levels of contamination (10, 50, 100, 200 and 500 EPG).

## Methods

### Study design and sampling

The study was conducted in two laboratories, one in Belgium and one in Italy.

In Belgium, GIN-positive and negative faecal samples were collected from Belgian Blue cattle stabled at the experimental farm of the Faculty of Veterinary Medicine (Ghent University). Positive samples were collected from calves (6 months-old) experimentally infected with 50,000 third-stage larvae (L3) of *Ostertagia ostertagi* (*n* = 2 calves) or *Cooperia oncophora* (*n* = 2 calves), whilst negative samples were collected from uninfected adult (> 24 months-old) housed cattle (*n* = 2 calves).

In Italy, GIN positive and negative faecal samples were collected from Podolian adult cattle (> 24 months-old) in a commercial farm located in the Salerno Province, Campania region. Positive samples were collected from cattle at pasture, naturally infected by different species of GINs, whilst negative samples were collected from stabled cattle. Each sample was analysed in five replicates by the FLOTAC basic technique [15] with an analytical sensitivity of 1 egg per gram (EPG) of faeces to determine the presence/absence of GIN eggs.

Both in Belgium and in Italy, the positive cattle were used as donors for the extraction of GIN eggs from faeces, using a mass recovery method, i.e. a method that employs 4 sieves of different mesh size (1 mm, 250 μm, 212 μm and 38 μm) in order to separate the eggs from the faeces, as described in Bosco et al. [12]. Eggs were recovered by washing the 38 μm sieve with tap water, and centrifuging the eluate for 3 min at 4000× *g*. To concentrate the GIN eggs, the supernatant was removed by a water pump and the pellet was resuspended in 5 ml of a 40% sucrose solution.

After centrifugation for 3 min at 4000×*g*, the supernatant was transferred to a new tube, diluted with an equal volume of tap water and centrifuged again for 3 min at 4000×*g*. The supernatant was removed to reduce the final volume of the egg preparation to 5 ml. Then, 10 aliquots of 0.1 ml each were taken, after a thorough homogenization of egg preparation into two tubes for 10 times (avoiding foam formation) for each aliquot to provide precise counting of eggs [12]. Finally, the number of eggs was counted at 100× magnification.

The egg suspensions were added to five confirmed negative faecal samples of 200 g each to obtain five samples with different EPG levels: 10, 50, 100, 200 and 500 EPG. Each sample was analysed, using saturated sodium chloride solution (specific gravity = 1.200), by two FEC techniques: Mini-FLOTAC [8] and a modified McMaster [16] technique at two reading levels, i.e. grids and chambers. In total, 12 replicates were used for each method and for each EPG level.

From each homogenised faecal sample, for each EPG level, 60 g were weighed for the Mini-FLOTAC technique (5 g for each replicate; dilution ratio = 1:10; reading volume = 2 ml; analytical sensitivity = 5 EPG) and 36 g for the McMaster technique (3 g for each replicate), reading the two grids (dilution ratio = 1:15; reading volume = 0.30 ml; analytical sensitivity = 50 EPG) and the two chambers (reading volume = 1 ml; analytical sensitivity = 15 EPG). All samples were prepared, analysed and read at 100× magnification by the same expert operator in Belgium and Italy.

### Coprocultures

In Italy, a pooled faecal culture was performed in order to identify the nematodes to the genus level, following the protocol described in MAFF [16]. Developed third-stage larvae (L3) were identified using the morphological keys proposed by van Wyk & Mayhew [17]. Identification and percentages of nematodes by genus were conducted on 100 L3; if a sample had 100 or less L3 present, all larvae were identified.

### Statistical analysis

EPG values for each technique and for each GIN infection level were calculated by multiplying the raw counts by the appropriate multiplication factor (e.g. 5 for Mini-FLOTAC, 50 for McMaster grids and 15 for McMaster chambers) and then, the mean of the replicate counts for each sample was calculated. The sensitivity of each method was estimated using the following formula: [(total number of positive samples observed/12, i.e. total number of replicate spiked samples performed for each method and for each level of contamination) × 100].

To evaluate the precision of each method, a coefficient of variation (CV) [(standard deviation/mean egg count) × 100] was calculated for each set of replicate counts for each method and level of EPGs. Furthermore, the accuracy of each method was determined by the percentage (%) of egg recovery calculated for each level of contamination, using the following formula: % egg recovery = [(observed FEC/ true FEC) × 100]. Boxplots (indicating median, percentiles and outliers) were used to show the precision and accuracy of each technique for each of the five levels of egg contamination. The non-parametric Kruskal-Wallis test with Dunn’s *post-hoc* test were used to compare all the observed values to the true FEC value for each technique and for each level of contamination.

Finally, a logistic regression model was developed in order to evaluate the predicted accuracy of each technique. The Mann-Whitney comparison test was used to compare the GIN egg recovery rates by Mini-FLOTAC and McMaster (reproducibility) in the two different laboratories (Belgium and Italy) using different samples from cattle experimentally (Belgium) or naturally infected by different GIN species (Italy), of different ages (calves in Belgium *vs* adult cattle in Italy) and breed (Belgian Blue *vs* Podolian).

All statistical analyses were performed in GraphPad Prism v.8 (Graph Pad Software, San Diego, CA, USA). Significance testing was set at *P *< 0.05.

## Results

**Fig. 1:**
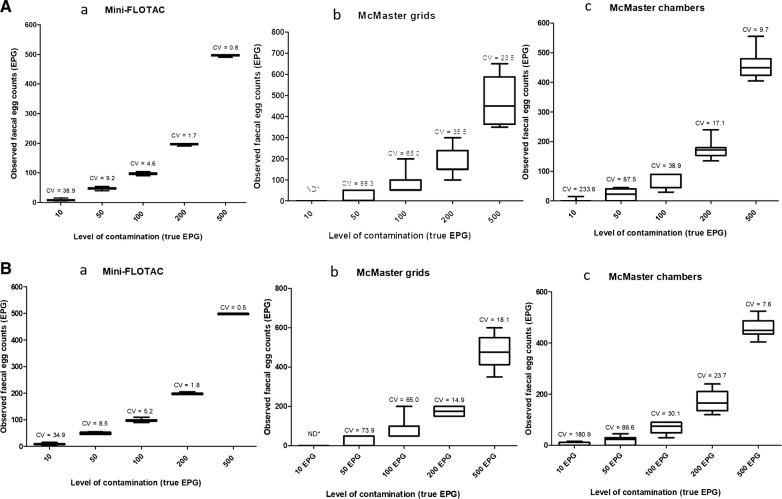
Boxplots of observed faecal egg counts (y axis) with Mini-FLOTAC technique (**a**), McMaster grids (**b**) and McMaster chambers (**c**) for the five EPG levels of contamination in Belgium (**A**) and in Italy (**B**)

A total of 360 counts were performed. The Mini-FLOTAC technique showed a sensitivity of 100% at all the EPG levels whilst the McMaster technique (reading either grids or chambers) showed a sensitivity of 100% only for levels ≥ 100 EPG. Below 100 EPG the sensitivity of McMaster grids and chambers ranged between 0–66.6%. Figure 1 and Table 1 show the boxplot, the precision (CV %) and the accuracy (%) of the observed mean EPG for each country at each level of egg contamination for Mini-FLOTAC, McMaster grids and chambers. The boxplots of the Mini-FLOTAC technique (Fig. 1) were very narrow for each contamination level, thus indicating a high precision compared to the McMaster grids and chambers. CVs for McMaster grids and chambers were higher than those of Mini-FLOTAC, especially for low counts.

**Table 1:** Mean accuracy (%) of Mini-FLOTAC and McMaster (grids and chambers) at the different EPG levels resulting from the experiment performed in Belgium and in Italy

FEC method	10 EPG	50 EPG	100 EPG	200 EPG	500 EPG
Belgium					
Mini-FLOTAC	95.8	96.7	97.9	98.5	99.4
McMaster grids	0	58.3	87.5	87.5	95.0
McMaster chambers	25.0	42.5	68.8	88.2	92.0
Italy					
Mini-FLOTAC	95.8	98.3	99.2	99.6	99.5
McMaster grids	0	66.7	87.5	87.5	95.8
McMaster chambers	37.5	40.0	68.8	84.4	91.3

**Fig. 2:**
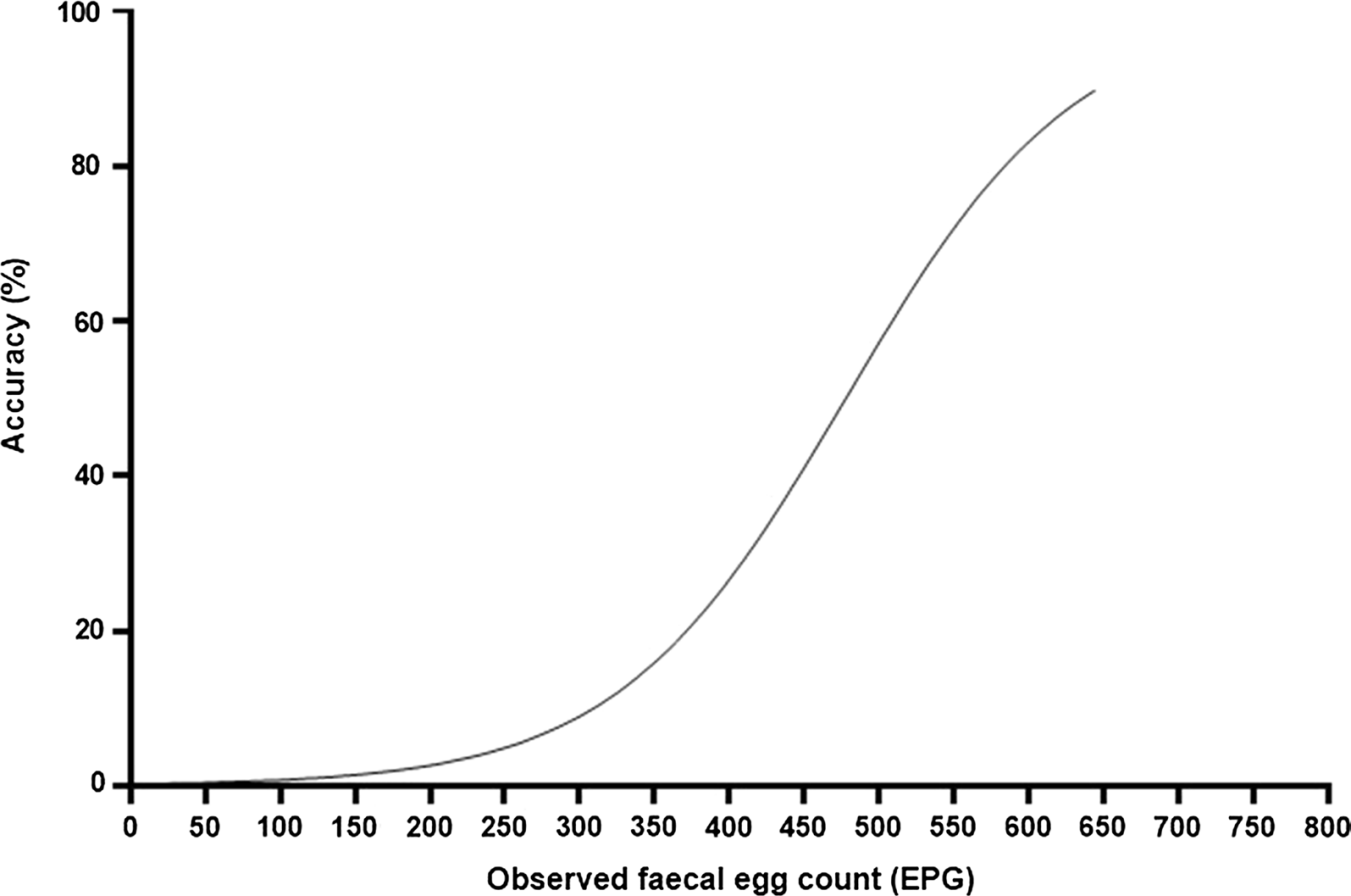
The predicted accuracy derived from the logistic regression for McMaster technique at the grid level

The Kruskal-Wallis test showed that there were significant differences for McMaster grids and McMaster chambers between observed and true EPG values at 10 EPG (Kruskal-Wallis H-test: *χ*^2^ = 57.8, *df*  =  3, *P *< 0.0001), 50 EPG (Kruskal-Wallis H-test: *χ*^2^ = 44.5, *df*  =  3, *P *< 0.0001), 100 EPG (Kruskal-Wallis H-test: *χ*^2^ = 43.4, *df* =  3, *P *< 0.0001) and 200 EPG (Kruskal–Wallis H-test: *χ*^2^ = 17.8, *df* = 3, *P *< 0.0001) levels of contamination, whilst at the level of 500 EPG, only McMaster grids (Kruskal–Wallis H-test: *χ*^2^ = 20.4, *df * =  3, *P *> 0.05) showed no statistically significant difference between observed and true values. This finding was confirmed by the results of the logistic regression (Fig. 2), the McMaster grids showing a low predicted accuracy related to low FEC, whilst becoming more accurate only when the FEC level increased, i.e. at 500 EPG.

Finally, the McMaster chambers had a low accuracy at all the levels of contamination, whilst the Mini-FLOTAC did not show any significant difference between the observed and the true values at all the EPG contamination levels.

*P*-values from the Mann-Whitney test ranged from 0.215 to 0.977 (from 10 to 500 EPG levels), showing that there was no significant difference of GIN egg recovery rates (either using Mini-FLOTAC or McMaster) obtained in Belgium and in Italy.

### Coprocultures

The following GIN genera were detected in the naturally infected samples collected from cattle in Italy: *Cooperia* (52%), *Trichostrongylus* (37%), *Ostertagia* (7%) and *Haemonchus* (4%).

## Discussion

The comparison between Mini-FLOTAC and McMaster for GIN FEC in cattle showed that Mini-FLOTAC had a higher sensitivity and accuracy and a lower CV than the McMaster technique (grids and chambers). Interestingly, McMaster grids showed higher FECs than McMaster chambers for all levels of contamination (10, 50, 100, 200 and 500 EPG). As described in Cringoli et al. [18] and Bosco et al. [12] it may be due to the tendency of eggs, during the flotation, to concentrate in the center of the McMaster slide, with a consequent overestimation of EPGs, especially at low egg counts. Moreover, McMaster showed no statistically significant difference between observed and true EPG only at 500 EPG and at grids level of reading. These results, therefore, showed that the McMaster is not a satisfactory method at low EPG levels, especially when the FECRT is used to evaluate the efficacy of anthelmintics and to detect anthelmintic resistance [7, 12, 19].

In this study, the mean percentage of recovery of GIN eggs with Mini-FLOTAC was very high, i.e. 98.1%. This result is in agreement with Godber et al. [10] and Bosco et al. [12] who found a 100% recovery rate of GIN eggs in sheep spiked faeces. The study by Paras et al. [7] showed a 70.9% recovery rate of cattle GIN eggs by Mini-FLOTAC that was higher than the values by other techniques (30.9% by modified Wisconsin and 55.0% by McMaster), but lower than the value detected in our study (98.1%). Similarly, Noel et al. [20] found a 42.6% recovery rate of equine strongyle eggs by Mini-FLOTAC, that was higher than the value from the McMaster technique (23.5%). In the study on equine faecal samples by Napravnikova et al. [21] the accuracy of Mini-FLOTAC was 74.2% (lower than McMaster) for strongyles and 90.3% (higher than McMaster) for ascarids. Finally, Scare et al. [22] compared an automated FEC using a smartphone with Mini-FLOTAC and McMaster and found a higher accuracy by Mini-FLOTAC (64.5%) compared to McMaster (21.7%) and the smartphone system (32.5%).

As described in Cringoli et al. [8] and in Norris et al. [23], the procedure of egg isolation and faeces contamination, as well as the choice of the flotation solution may influence the recovery rates of a technique in any egg-spiking experiment. These factors may have contributed to the high recovery rate of GIN eggs in cattle using Mini-FLOTAC in the present study as argued below. First, with regard to the spiking procedure, in our study we spiked cattle faeces with GIN eggs obtained from cattle that were either experimentally (in Belgium) or naturally (in Italy) infected with GINs. This could explain the higher accuracy compared to the findings by Paras et al. [7] where eggs isolated from goat faeces were used to contaminate cattle faeces. In support of our hypothesis, a recovery rate only of 91.0% was obtained by Bosco et al. [12] when GIN eggs from sheep were added to horse faeces. Secondly, the choice of the flotation solution is very important, as it may influence the performance of the technique and therefore its precision and accuracy [8].

In different studies it has been shown that sodium chloride (specific gravity = 1.20) was the best flotation solution for GIN FEC and it is recommended when using the Mini-FLOTAC technique [8]. Therefore, the low recovery rates found in the above mentioned studies could be due to the inappropriateness of the flotation solutions (i.e. sodium nitrate with a specific gravity = 1.25–1.30 [7]; and glucose-NaCl flotation medium with a specific gravity = 1.24–1.28 [20–22].

In our study, CVs of Mini-FLOTAC were lower than the CVs of McMaster grids and chambers for all levels of contamination as reported also in other studies [7, 10, 12, 13, 20–22, 24]. Furthermore, CVs for McMaster chambers were lower than those obtained with McMaster grids, in agreement with Godber et al. [10] and Bosco et al. [12]. To support these findings, Levecke et al. [25] and Torgerson et al. [26] showed that precision increases when analytical sensitivity increases; with the McMaster technique, the variance of EPG estimates between repeated samples of the same faecal sample is inflated, due to the multiplication factor when transforming the raw counts in EPG [26]. Moreover, in the present study CVs were lower at higher levels of contamination for all the techniques, as also reported by Mes et al. [27] and Das et al. [28], the precision increases when the EPG in faecal sample increases.

## Conclusions

Since the sensitivity, precision and accuracy of a FEC depend on many factors, it is very important to establish precise standard operating procedures (SOPs) for FEC techniques, including the flotation solution to use. In fact, it is surprising that diagnostic and research laboratories around the world use different protocols of FEC techniques for their activities. In this regard, research priorities should include the development of more scalable, reliable, less labour-intensive systems for parasite egg counts for both pen-side and laboratory use [5], including methods of automated sample processing and image analysis [29] as indicated in the STAR-IDAZ (https://www.star-idaz.net) diagnostic road map for research on helminths and anthelmintic resistance.

## Data Availability

All data generated or analysed during this study are included in this published article. The datasets used and/or analysed during the present study available from the corresponding author upon reasonable request.

## Abbreviations

AR: anthelmintic resistance
CV: coefficient of variation
EPG: egg per gram of faeces
FEC: faecal egg count
FECRT: faecal egg count reduction test
GIN: gastrointestinal nematode
L3: third-stage larvae
SOPs: standard operating procedures
